# The Impact of Time Interval Between First Extubation and Reintubation on Bronchopulmonary Dysplasia or Death in Very Low Birth Weight Infants

**DOI:** 10.3389/fped.2022.867767

**Published:** 2022-04-25

**Authors:** Jing Li, Jing Zhang, Qingfei Hao, Ziyun Shen, Yanna Du, Haoming Chen, Xiuyong Cheng

**Affiliations:** Department of Neonatology, The First Affiliated Hospital of Zhengzhou University, Zhengzhou, China

**Keywords:** very low birth weight infant, extremely low birth weight infant, reintubation, bronchopulmonary dysplasia, death

## Abstract

**Objective:**

To explore the association between time from first extubation to reintubation and moderate-to-severe bronchopulmonary dysplasia (BPD) or death in very low birth weight infants.

**Study Design:**

Infants weighing <1,500 g at birth, requiring mechanical ventilation, and undergoing their initial extubation were retrospectively included from January 2014 to December 2021. They were divided into the moderate-to-severe BPD/death group and the comparison group according to the incidence of moderate-to-severe BPD or death. We defined time to reintubation as the time interval between first extubation and reintubation. In a stepwise multivariate logistic regression analysis, we examined the association between time to reintubation and moderate-to-severe BPD/death using different observation windows after initial extubation (24-h intervals).

**Results:**

A total of 244 infants were recruited, including 57 cases in the moderate-severe BPD/death group and 187 cases in the comparison group, and 93 (38.1%) cases were reintubated at least one time after their first extubation. Univariate analysis showed that reintubation rates within different observation windows in the moderate-to-severe BPD/death group were statistically significantly (*p* < 0.05) higher than those in the comparison group. Multivariate regression analysis showed that reintubation within observation windows 48 h or 72 h post-extubation was an independent risk factor in moderate-to-severe BPD/death and death, but not moderate-to-severe BPD. When the time window was 48 h, the probability of moderate-to-severe BPD/death [odds ratio (OR): 3.778, 95% confidence interval (CI): 1.293–11.039] or death (OR: 4.734, 95% CI: 1.158–19.354) was highest. While after extending the observation window to include reintubations after 72 h from initial extubation, reintubation was not associated with increased risk of moderate-to-severe BPD and/or death.

**Conclusions:**

Not all reintubations conferred increased risks of BPD/death. Only reintubation within 72 h from initial extubation was independently associated with increased likelihood of moderate-to-severe BPD/death and death in very low birth weight infants, and reintubation within the first 48 h post-extubation posed the greatest risk.

## Introduction

Studies have shown that nearly 47% of very low birth weight and 60–70% of extremely low birth weight infants had extubation failure during hospitalization and required reintubation (1, 2). Extubation failure is defined as reintubation within a specified time window from extubation. Currently, there is a lack of a uniform definition of extubation failure in neonates. According to a systematic review, the duration of the observation window used to define extubation failure varied a lot, ranging from a few hours to several weeks after extubation (3).

Recently, a subgroup analysis of a randomized controlled study has found that reintubation within 5 days after initial extubation was independently associated with increased mortality and/or respiratory morbidities in extremely preterm infants (4). In contrast, a subgroup analysis of another randomized controlled study found that reintubation within 7 days after initial extubation was not an independent risk factor in the occurrence of BPD and BPD/death (5). This suggests that reintubation within hours of first extubation may have different clinical significance from reintubation days or weeks later. Therefore, we sought to investigate associations between time to reintubation and outcomes of moderate-to-severe BPD or death in a retrospective cohort of very low birth weight infants.

## Materials and Methods

### Selection of Patients

We conducted a retrospective cohort study of infants born in our hospital and cared for at our Neonatal Intensive Care Unit (NICU) between September 2014 and December 2021. Premature infants with birth weight <1,500 g and gestational age <32 weeks, requiring mechanical ventilation and undergoing their first extubation were included. All treatment decisions, including weaning from mechanical ventilation, extubation, and reintubation, were made by the treating team independent of the study. Infants were intubated or reintubated under the following circumstances: (1) frequent apnea, ineffective by drug or CPAP intervention; (2) infants with RDS need to be treated with PS; (3) FiO_2_ >0.6~0.7, PaO_2_ <50~60 mmHg or TcSO_2_ <85% (except cyanotic congenital heart disease); (4) PaCO_2_ >60~65 mmHg, accompanied by persistent acidosis (pH <7.20); and (5) under general anesthesia (6). Preterm infants received extubation using the following criteria: (1) the primary disease of the infants improved, the infection was basically controlled, and the general condition (including stable breathing, stable oxygen saturation, and blood gas analysis) was good; (2) peak inspiratory pressure ≤ 18-cm H_2_O, positive end-expiratory pressure at 2 to 4-cm H_2_O, frequency of ventilator <10 breaths/min, a fraction of inspired oxygen (FiO_2_) ≤ 0.4, and arterial blood gas analysis were normal (6). Exclusion criteria were high-frequency ventilation in the initial intubation, extubation directly to low flow nasal cannula or no respiratory support, major heart defects or congenital anomalies, and deaths or discharge prior to initial extubation.

### Exposure

For each infant with extubation failure, we calculated “time to reintubation” based on the time interval between the date and the time of the initial extubation and the date and the time of reintubation. The reintubation rates of the infants within different observation windows were determined. Each observation window was 24-h intervals, thus, creating a wide range of exposures, ranging from “reintubation within 24 h” to “reintubation at any time during NICU hospitalization” after initial extubation.

### Outcomes of Interest

The primary outcome was the composite outcome of moderate-to-severe BPD or death. Moderate-to-severe BPD was defined as an oxygen requirement (>21% FiO_2_) for at least 28 days, and the need for any type of respiratory support at 36 weeks post-menstrual age (7). The secondary outcomes were moderate-to-severe BPD and death. The infants who developed moderate-to-severe BPD or/and death were retrospectively divided into the BPD/death group and the remaining were included in the comparison group.

### Covariates

Potential confounding variables involving maternal and neonatal clinical characteristics were included in the statistical model. They were divided into four aspects as follows: (1) maternal variables: delivery mode, multiple pregnancies, antenatal steroids, gestational diabetes, and gestational hypertension; (2) neonatal demographic variables: gestational age (GA), birth weight (BW), male sex, small for gestational age (8), 5-min Apgar score, surfactant use, caffeine use, postnatal intravenous steroids, pre-extubation blood gas (pH and PaCO_2_) and FiO_2_, and post-extubation respiratory support modes. Post-extubation non-invasive respiratory support modes included continuous positive airway pressure (CPAP), nasal intermittent positive pressure ventilation (NIPPV), non-invasive high-frequency oscillatory ventilation (NIHFO); (3) neonatal comorbidities: patent ductus arteriosus, pneumonia, culture-proven postnatal infection (bloodstream, cerebrospinal fluid, or urine), severe necrotizing enterocolitis (requiring surgical intervention) (9), and severe retinopathy of prematurity (ROP) (10).

### Statistical Analysis

All analyses were conducted using SPSS 22.0 software. Continuous variables are reported as mean and standard deviation or medians and interquartile ranges [(IQR): 25th−75th percentile], and categorical variables as frequencies and percentages. Patient characteristics were compared between groups, as well as infants reintubated and those never reintubated, using the χ^2^-test and Fisher's exact test (for categorical variables) or Student's *T*-test and Wilcoxon rank-sum test (for continuous variables). We used stepwise, multivariate logistic regression to estimate the association between time to reintubation and the outcomes of interest. The cumulative proportion of the infants that developed BPD/death was determined as a function of time to reintubation. Clinically relevant variables that were significantly different with *p* < 0.05 between groups were included in the multivariable logistic regression. Adjusted odds ratios (aOR) and 95% confidence intervals (CI) were then calculated.

## Results

**Figure 1:**
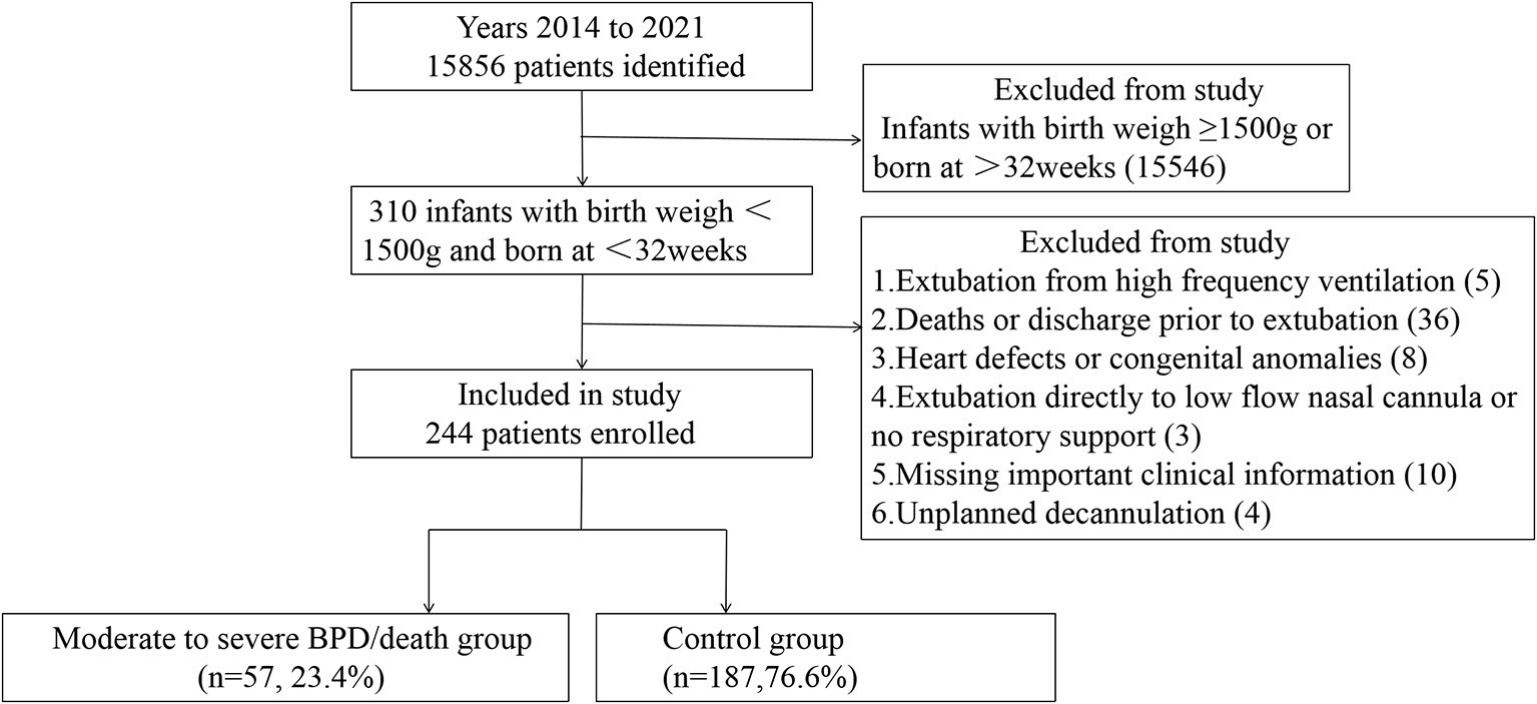
A flow chart of this study.

A total of 15,856 preterm infants were born and admitted to our NICU for the first time during the study period, and 244 of them were included in the study (Figure 1). In the moderate-to-severe BPD/death group, there were 26 infants with moderate-to-severe BPD, 22 deaths, and 9 newborns with both moderate-to-severe BPD and death. The infants who developed moderate-to-severe BPD/death (gestational age: 28.7 ± 1.6; birth weight: 1025.6 ± 178.6 g) were significantly smaller and more immature at birth compared with those in the comparison group (gestational age: 29.3±1.4; birth weight: 1189.8 ± 479.4 g). By discharge from the NICU, the infants with moderate-to-severe BPD/death received a longer duration of mechanical ventilation and had significantly more mechanical ventilation courses, transfusions, higher rates of infection compared with the infants in the comparison group (Table 1). At the time of extubation, they also had more days on mechanical ventilation, higher PaCO_2_, and lower pH in blood gas analysis. There were no significant differences between groups in the mode of non-invasive respiratory support used immediately after extubation. The cumulative reintubation rates of the infants in the BPD/death group were higher than those in the comparison group in all different observation windows (Table 2). Of these 244 infants included in the analysis, 93 (38.1%) were reintubated at least one time after their first extubation.

**Table 1:** Comparison of clinical characteristics between the bronchopulmonary dysplasia (BPD)/death group and the comparison group.

**Variables**	**Comparison group (*n* = 187)**	**BPD/death group (*n* = 57)**	***P*-value**
Neonatal demographics
Gestational age, week	29.3 ± 1.4	28.7 ± 1.6	0.014
Birth weight, g	1189.8 ± 479.4	1025.6 ± 178.6	0.012
Small for gestational age	30 (20.3)	19 (33.3)	0.042
Male sex	119 (63.6)	35 (61.4)	0.624
Apgar score 5 min	8 (7.0, 9.0)	8.0 (7.0, 8.0)	0.030
Intubation in delivery room	86 (46.0)	34 (59.6)	0.071
Surfactant ≥2 times	38 (20.3)	21 (36.8)	0.011
Postnatal steroids	39 (20.9)	11 (19.3)	0.799
Caffeine	172 (92.0)	52 (91.2)	1.000
Outcomes by discharge
Number of transfusions	2.5 (1.0, 4.0)	4.0 (2.0, 6.0)	0.004
Cumulative MV days	2.5 (1.1, 4.8)	6.9 (2.7, 18.4)	<0.001
Number of MV courses	1.0 (1.0, 2.0)	2.0 (1.0, 3.0)	<0.001
Patent ductus arteriosus	31 (16.6)	15 (26.3)	0.100
Postnatal infection	20 (10.7)	18 (31.6)	<0.001
Necrotizing enterocolitis	5 (2.7)	2 (3.5)	1.000
Retinopathy of prematurity	13 (7.0)	6 (10.5)	0.549
Maternal demographics
Cesarean section	143 (76.5)	41 (71.9)	0.486
Multiple births	51 (27.3)	15 (26.3)	0.887
Antenatal steroids	113 (60.4)	33 (57.9)	0.733
Gestational hypertension	98 (52.4)	31 (54.4)	0.101
Gestational diabetes	29 (15.5)	4 (7.0)	0.101

*Data are presented as counts (percentages) or median (interquartile range); MV, mechanical ventilation*.

**Table 2:** Comparison of respiratory variables between the BPD/death group and the comparison group.

**Variables**	**Comparison group (*n* = 187)**	**BPD/death group (*n* = 57)**	***P*-value**
Pre-extubation
Postnatal age, w	29.7 (28.7, 31.0)	29.3 (28.3, 30.7)	0.075
PH	7.4 (7.4, 7.5)	7.4 (7.3, 7.5)	0.002
PaCO_2_, mmH_2_O	32.0 (29.0, 41.0)	37.0 (32.0, 44.5)	0.005
FiO_2_, mL/L	25.0 (25.0, 30.0)	30.0 (25.0, 30.0)	0.487
MV, d	1.6 (0.9, 3.3)	6.9 (2.7, 18.4)	0.017
Post-extubation
CPAP	124 (66.3)	36 (63.2)	0.661
NIPPV	43 (23.0)	10 (17.5)	0.382
NIHFO	20 (10.7)	11 (19.3)	0.088
Reintubation in different observation windows			
≤ 24 h	17 (9.1)	16 (28.1)	<0.001
≤ 48 h	25 (13.4)	26 (45.6)	<0.001
≤ 72 h	28 (15.0)	28 (49.1)	<0.001
≤ 5 d	35 (18.7)	30 (52.6)	<0.001
≤ 7 d	39 (20.9)	31 (54.4)	<0.001
≤ 14 d	44 (23.5)	34 (59.6)	<0.001
≤ 21 d	46 (24.6)	38 (66.7)	<0.001
Anytime	53 (28.3)	40 (70.2)	<0.001

*Data are presented as counts (percentages), mean (standard deviation), or median (interquartile range); BPD, bronchopulmonary dysplasia; PaCO_2_, partial pressure of arterial blood carbon dioxide; FiO_2_, volume fraction of inhaled oxygen; MV, invasive mechanical ventilation; CPAP, continuous positive airway pressure ventilation; NIPPV, intermittent positive nasal pressure ventilation; NIHFO, non-invasive high frequency oscillatory ventilation*.

Table 3 shows adjusted relationships between time to reintubation after extubation and outcomes for different observation windows. Adjustments were made for variables that were significantly different with *p* < 0.05 between groups in multivariate models. Reintubation within observation window 48 or 72 h post-extubation was associated with significantly greater odds of the primary outcome of moderate-to-severe BPD/death and the secondary outcome of death. The aOR appeared to be highest for reintubations occurring within 48 h after extubation. However, extending the window to include reintubations after 72 h post-extubation led to non-significant associations. Reintubation within different observation windows was not an independent risk factor in the development of moderate-to-severe BPD. Moreover, birth weight, total mechanical ventilation days, and pneumonia remained significantly associated with the development of moderate-to-severe BPD/death when choosing different observation windows to define extubation failure.

**Table 3:** Adjusted odds of BPD/death, BPD, and death as a function of reintubation within different observation windows after extubation.

**Outcomes/time of reintubation**	**OR**	**95%CI**	***P*-value**
Moderate-to-severe BPD/death
**≤**24 h	2.004	[0.669, 6.003]	0.215
**≤**48 h	3.778	[1.293, 11.039]	0.015
**≤**72 h	3.268	[1.127, 9.471]	0.029
**≤**5 d	2.115	[0.710, 6.304]	0.179
**≤**7 d	1.967	[0.664, 5.830]	0.222
**≤**14 d	1.231	[0.412, 3.680]	0.710
**≤**21 d	1.864	[0.622, 5.584]	0.266
Anytime	1.557	[0.481, 5.036]	0.460
Moderate-to-severe BPD
**≤**24 h	1.523	[0.451, 5.147]	0.498
**≤**48 h	1.529	[0.457, 5.114]	0.490
**≤**72 h	1.714	[0.546, 5.374]	0.356
**≤**5 d	1.813	[0.573, 5.731]	0.311
**≤**7 d	1.501	[0.482, 4.678]	0.484
**≤**14 d	0.641	[0.194, 2.120]	0.466
**≤**21 d	0.831	[0.240, 2.875]	0.771
Anytime	0.826	[0.213, 3.203]	0.782
Death
**≤**24 h	2.246	[0.533, 9.460]	0.270
**≤**48 h	4.734	[1.158, 19.354]	0.030
**≤**72 h	4.321	[1.081, 17.277]	0.038
**≤**5 d	2.483	[0.588, 10.493]	0.216
**≤**7 d	2.885	[0.702, 11.859]	0.142
**≤**14 d	2.442	[0.590, 10.112]	0.218
**≤**21 d	2.730	[0.641, 11.630]	0.174
Anytime	1.527	[0.317, 7.358]	0.598

*BPD, bronchopulmonary dysplasia*.

To understand why the time to reintubation was independently correlated with increased risk of moderate-to-severe BPD/death and deaths, but not moderate-to-severe BPD, we performed a *post-hoc* analysis of the reintubation timing among the infants in the moderate-to-severe BPD/death group. Reintubation timing of the infants, who developed moderate-to-severe BPD or death, is shown in Table 4. There was a total of 31 infant deaths in the cohort: 19 (61.3%) were reintubated within 72 h; 6 infants (19.4%) never required reintubation. While among the 35 infants who developed moderate-to-severe BPD, only 16 (45.7%) were reintubated within 72 h; up to 11 infants (31.4%) never required reintubation. Lastly, given the high risk of moderate-to-severe BPD/death and death associated with reintubations within the first 72 h post-extubation, we performed a second *post-hoc* evaluation to determine whether some differences existed in these infants when compared with those reintubated between 73 h and 7 days. Table 5 shows that no statistically significant differences could be detected, except for a significantly increased proportion of the infants who received surfactant for more than one time among those reintubated within 72 h post-extubation.

**Table 4:** Reintubation timing of infants developed moderate-to-severe BPD or death.

**Timing of reintubation**	**Moderate-to-severe BPD, *n* (%)**	**Death, *n* (%)**
0~24 h	11 (31.4)	9 (29.0)
24~48 h	4 (11.4)	8 (25.8)
48~72 h	1 (2.9)	2 (6.5)
72 h~5 d	2 (2.7)	1 (3.2)
5~7 d	0	1 (3.2)
7~14 d	1 (2.9)	3 (9.7)
14~21 d	3 (8.6)	1 (3.2)
21 d~anytime	2 (2.7)	0
Never reintubated	11 (31.4)	6 (19.4)

**Table 5:** Characteristics of infants reintubated within 72 h after extubation vs. those reintubated at 73 h−7 days after extubation.

**Variables**	**Reintubated within 72 h (*n* =56)**	**Reintubated at 73 h−7 days (*n* =31)**	***P*-value**
Demographics
Gestational age, w	28.4 (27.4, 29.3)	29.1 (27.9, 30.0)	0.212
Birth weight, g	1038.4 ± 172.6	1126.4 ± 195.2	0.101
Small for gestational age	15 (26.8)	5 (35.7)	0.741
Antenatal corticosteroids	29 (51.8)	9 (64.3)	0.401
Apgar score 5 min ≤ 5	9 (16.1)	0	0.246
Intubation in the delivery room	33 (58.9)	6 (42.9)	0.279
Surfactant ≥2 times	24 (42.9)	2 (14.3)	0.048
Postnatal steroids	23 (41.1)	2 (14.3)	0.061
Caffeine	49 (87.5)	13 (92.9)	0.925
Postnatal infection	11 (19.6)	2 (14.3)	0.939
Pneumonia	28 (50)	8 (57.1)	0.632
Severe necrotizing enterocolitis	0	7 (7.1)	0.200
Pre-extubation
Postnatal age	28.9 (28.0, 30.0)	29.6 (28.6, 31.3)	0.071
MV, d	1.8 (1.0, 3.4)	2.1 (1.0, 4.6)	0.849
PH	7.4 ± 0.1	7.4 ± 0.1	0.111
PaCO_2_, mmH_2_O	39 ± 9.7	35.4 ± 10.9	0.224
Hb, g/L	146.4 ± 30.0	146.6 ± 32.1	0.987

*Data are presented as counts (percentages), mean (standard deviation), or median (interquartile range); MV, invasive mechanical ventilation*.

## Discussion

In this single-center retrospective study, we found that reintubation within 72 h after extubation independently modulated the probability of the outcome of moderate-to-severe BPD/death and death, conferring the greatest risk when it occurred within 48 h post-extubation. Reintubations after 72 h from initial extubation are not associated with the development of moderate-to-severe BPD or death. These results suggest that early reintubations may predict an increased risk of BPD/death and death in very low birth weight infants.

In adults, it is generally accepted that extubation failure is associated with increased mortality (11). In neonates, there are conflicting opinions as to whether reintubation is associated with the development of BPD and/or death. An international survey found that 47% of experts considered extubation failure as an independent risk factor in increased morbidities rates and mortality in preterm infants (12). Three recent large studies analyzing the associations between reintubation and respiratory morbidities or death in extremely preterm infants also had partially inconsistent findings (1, 4, 5). One of the reasons was that each study used a different definition of extubation failure for evaluation. Shalish et al. (13), therefore, investigated associations between time to reintubation and neonatal outcome of BPD or death. However, the quite wide 95% CIs indicated a high uncertainty regarding the true strength of the associations observed in their study. Also, mortality was only evaluated as part of a composite outcome rather than separately due to the small number of deaths. Thus, we sought to explore these issues systematically using our study cohort.

After correcting for confounding factors, including duration of mechanical ventilation, we demonstrated that the probability of moderate-to-severe BPD/death was highest for the infants reintubated within 48 h after extubation, which was consistent with Shalish et al.'s study (13). Our findings validated and extended the results of the previous study (13). According to their results, the longer the observation window to include later reintubations, the weaker the correlations with BPD/death became, and ultimately non-significant when included all reintubations before discharge. While, in this cohort, we found that only reintubations within 72 h from extubation were independently associated with an increased risk of moderate-to-severe BPD/death and the secondary outcome of death. But extending the observation window to include reintubations after 72 h resulted in totally non-significant correlations. Our observations above validated the hypothesis in the previous study that, although reintubation within broader observation windows (e.g., 7 or 14 days) is associated with independently increased risk of BPD/death, the significance may be attributed to earlier reintubations (13).

Careful scrutiny of the reintubations, as well as reviewing previous literature, leads us to speculate about some plausible mechanisms that may have played a role in the findings above. Firstly, the time of extubation failure simply reflected the physiological immaturity of the newborn's systems and illness severity of those neonates. In this study, a total of 56 infants were reintubated within the first 72 h after extubation, and 28 (50%) of them developed BPD or/and death. While among the 14 infants reintubated between 73 h and 7 days, 3 infants (21.4%) developed BPD or/and death. The infants who failed extubation within the first 3 days were less mature, although insignificantly (*p* > 0.05), and more likely to require a second dose of surfactant compared with those reintubated from 73 h to 7 days from extubation (*p* < 0.05), suggesting that early extubation failure may be a marker of an inherently more immature and sicker group. Thus, the infants who were reintubated after 72 h were more likely to survive to discharge without BPD or/and death.

However, whether extubation failure merely signifies a poor prognosis or contributes to it has always been the issue. A prospective observational study of adults suggested that extubation failure can have a direct impact on patient outcomes independently of underlying illness severity (14). Other adult studies have shown that the pressure support provided by mechanical ventilation can significantly decrease pulmonary wedge pressure, work on breathing, and improve left ventricular function (15). However, whether evidence from these adult studies can be extrapolated to preterm newborns remains unknown. In a recent large cohort study, 5 min of spontaneous breathing trial (SBT) caused some form of cardiorespiratory instability in nearly 60% of preterm infants (16). Gupta et al. (17) reported that the respiratory status of the infants who failed extubation was worse at 24 and 72 h after reintubation when compared to pre-extubation levels, and it took many days to achieve pre-extubation levels after reintubation. In another retrospective study, the infants who failed extubation did not increase breathing frequency or work of breathing statistically significantly following a 3-min SBT compared to those infants with a successful extubation (17). The inability to compensate for the transiently increased cardiorespiratory load during a trial might be an indicator of cardiorespiratory function deterioration, which can lead to potentially catastrophic (sometimes fatal) consequences (18–20). Therefore, a reasonable assumption is that the infants required early reintubation (within 72 h) deteriorated clinically after initial extubation as a direct result of the removal of the ventilatory support provided during mechanical ventilation, such as atelectasis, apnea, and hemodynamic instability (21). The infants who were reintubated after 72 h were extubated at a more appropriate time, thus, they could compensate for the temporary increase in cardiorespiratory load for a short time after extubation. Further research investigating predictive models of extubation readiness and new interventions to prevent extubation failure is required to prevent such adverse events. Finally, it is well-known that non-respiratory-related reintubation rates increased as the time interval between initial extubation and reintubation increased (2). This may partly explain the disappearing associations between extubation failure and BPD/death when reintubations occurred after 72 h.

This study has some limitations. Our study is limited by confounders and biases inherent in a retrospective observational study design. Although we used logistic regression in our analysis, adjusting for several important variables known to increase BPD or death, residual confounding factors and biases may still be present. Lastly, the results may not apply to reintubations after two or more failed extubations or after accidental extubation because we only evaluated the impact of time to reintubations after initial extubation and excluded reintubations after unplanned decannulations.

In conclusion, reintubations within 72 h from initial extubation are independently associated with an increased risk of BPD/death and death in very low or extremely low birth weight infants, and this association is predominantly attributed to the infants reintubated within 48 h. Reintubations after 72 h post-extubation do not modulate the odds of BPD and/or death. Further studies are needed to validate these results prospectively and define the optimal timing of extubation to help reduce early extubation failures without increasing the total duration of mechanical ventilation.

## Data Availability Statement

The raw data supporting the conclusions of this article will be made available by the authors, without undue reservation.

## Author Contributions

JL and JZ conceived the idea. JL and QH analyzed the data. JL wrote the manuscript. JZ, QH, ZS, YD, HC, and XC reviewed and edited the manuscript. All authors contributed to the article and approved the submitted version.

## Conflict of Interest

The authors declare that the research was conducted in the absence of any commercial or financial relationships that could be construed as a potential conflict of interest. The handling editor YS declared past co-authorships with XC.

## Publisher's Note

All claims expressed in this article are solely those of the authors and do not necessarily represent those of their affiliated organizations, or those of the publisher, the editors and the reviewers. Any product that may be evaluated in this article, or claim that may be made by its manufacturer, is not guaranteed or endorsed by the publisher.

## References

[1] Jensen EA DeMauro SB Kornhauser M Aghai ZH Greenspan JS Dysart KC. Effects of multiple ventilation courses and duration of mechanical ventilation on respiratory outcomes in extremely low-birth-weight infants. JAMA Pediatr. (2015) 169:1011–7. 10.1001/jamapediatrics.2015.2401 26414549 PMC6445387

[2] Shalish W Kanbar L Keszler M Chawla S Kovacs L Rao S. Patterns of reintubation in extremely preterm infants: a longitudinal cohort study. Pediatr Res. (2018) 83:969–75. 10.1038/pr.2017.330 29389921

[3] Giaccone A Jensen E Davis P Schmidt B. Definitions of extubation success in very premature infants: a systematic review. Arch Dis Child Fetal Neonatal Ed. (2014) 99:F124–7. 10.1136/archdischild-2013-304896 24249694 PMC4025952

[4] Chawla S Natarajan G Shankaran S Carper B Brion LP Keszler M. Markers of successful extubation in extremely preterm infants, and morbidity after failed extubation. J Pediatr. (2017) 189:113–9. 10.1016/j.jpeds.2017.04.050 28600154 PMC5657557

[5] Manley BJ Doyle LW Owen LS Davis PG. Extubating extremely preterm infants: predictors of success and outcomes following failure. J Pediatr. (2016) 173:45–9. 10.1016/j.jpeds.2016.02.016 26960919

[6] Editorial Board Chinese Chinese Journal of Pediatrics; the Subspecialty Group of Neonatology the the Society of Pediatrics Chinese Chinese Medical Association and Editorial Board Chinese Journal of Pediatrics the Subspecialty Group of Neonatology the Society of Pediatrics Chinese Medical Association. Routine mechanical ventilation of neonates. Chin J Pediatr. (2015).53:327–30. 10.3760/cma.j.issn.0578-1310.2015.05.003 26080660

[7] Jobe AH Bancalari E. Bronchopulmonary dysplasia. Am J Respir Crit Care Med. (2001) 163:1723–9. 10.1164/ajrccm.163.7.2011060 11401896

[8] Fenton TR Kim JH. A systematic review and meta-analysis to revise the Fenton growth chart for preterm infants. BMC Pediatr. (2013) 13:59. 10.1186/1471-2431-13-59 23601190 PMC3637477

[9] Walsh MC Kliegman RM. Necrotizing enterocolitis: treatment based on staging criteria. Pediatr Clin North Am. (1986) 33:179–201. 10.1016/S0031-3955(16)34975-6 3081865 PMC7131118

[10] International Committee for the Classification of Retinopathy of Prematurity. The International Classification of Retinopathy of Prematurity revisited. Arch Ophthalmol. (2005) 123:991–9. 10.1001/archopht.123.7.991 16009843

[11] Jaber S Quintard H Cinotti R Asehnoune K Arnal JM Guitton C. Risk factors and outcomes for airway failure versus non-airway failure in the intensive care unit: a multicenter observational study of 1514 extubation procedures. Crit Care. (2018) 22:236. 10.1186/s13054-018-2150-6 30243304 PMC6151191

[12] Al-Mandari H Shalish W Dempsey E Keszler M Davis PG Sant'Anna G. International survey on periextubation practices in extremely preterm infants. Arch Dis Child Fetal Neonatal Ed. (2015) 100:F428–31. 10.1136/archdischild-2015-308549 26063193

[13] Shalish W Kanbar L Kovacs L Chawla S Keszler M Rao S. The impact of time interval between extubation and reintubation on death or bronchopulmonary dysplasia in extremely preterm infants. J Pediatr. (2019) 205:70–6. 10.1016/j.jpeds.2018.09.062 30404739

[14] Thille AW Harrois A Schortgen F Brun-Buisson C Brochard L. Outcomes of extubation failure in medical intensive care unit patients. Crit Care Med. (2011) 39:2612–8. 10.1097/CCM.0b013e3182282a5a 21765357

[15] Mahmood SS Pinsky MR. Heart-lung interactions during mechanical ventilation: the basics. Ann Transl Med. (2018) 6:349. 10.21037/atm.2018.04.29 30370276 PMC6186561

[16] Shalish W Kanbar L Kovacs L Chawla S Keszler M Rao S. Assessment of extubation readiness using spontaneous breathing trials in extremely preterm neonates. JAMA Pediatr. (2020) 174:178–85. 10.1001/jamapediatrics.2019.4868 31860014 PMC6990705

[17] Gupta D Greenberg RG Natarajan G Jani S Sharma A Cotten M. Respiratory setback associated with extubation failure in extremely preterm infants. Pediatr Pulmonol. (2021) 56:2081–6. 10.1002/ppul.25387 33819392

[18] Nakato AM Ribeiro DF Simao AC Da SR Nohama P. Impact of spontaneous breathing trials in cardiorespiratory stability of preterm infants. Respir Care. (2021) 66:286–91. 10.4187/respcare.07955 32994356

[19] Lemaire F Teboul JL Cinotti L Giotto G Abrouk F Steg G. Acute left ventricular dysfunction during unsuccessful weaning from mechanical ventilation. Anesthesiology. (1988) 69:171–9. 10.1097/00000542-198808000-00004 3044189

[20] Tobin MJ. Extubation and the myth of “minimal ventilator settings”. Am J Respir Crit Care Med. (2012) 185:349–50. 10.1164/rccm.201201-0050ED 22336673

[21] Odita JC Kayyali M Ammari A. Post-extubation atelectasis in ventilated newborn infants. Pediatr Radiol. (1993) 23:183–5. 10.1007/BF02013827 8332404

